# Lung Ultrasound Score at Hospital Discharge as a Predictor of Emergency Department Visit and Hospital Readmission in Patients With Acute Heart Failure

**DOI:** 10.7759/cureus.63051

**Published:** 2024-06-24

**Authors:** Aron H Ferreira, Daniel C Zoppi, Carlos H Miranda, Valdair F Muglia, Antonio Pazin-Filho

**Affiliations:** 1 Internal Medicine, Ribeirão Preto Medical School - University of São Paulo, Ribeirão Preto, BRA; 2 Internal Medicine, Ribeirão Preto Medical School - University of Sao Paulo, Ribeirão Preto, BRA; 3 Radiology, Ribeirão Preto Medical School - University of Sao Paulo, Ribeirão Preto, BRA

**Keywords:** lung ultrasound score, lung ultrasound, lus, heart failure, b-lines, acute heart failure

## Abstract

Purpose

The number of B-lines on lung ultrasound at hospital discharge in patients admitted with acute heart failure (AHF) is associated with poor outcomes. Assessing B-lines can be challenging to execute and replicate, depending on the clinical context. This study aims to determine whether the lung ultrasound score (LUS) at discharge predicts hospital readmission or emergency department (ED) visits in the 30 days after an AHF hospital admission.

Methods

We conducted an observational study at the medical ward of the emergency unit of the Clinics Hospital of the Ribeirao Preto Medical School, University of Sao Paulo, a tertiary university hospital in Ribeirao Preto, Sao Paulo, Brazil, where consecutive adults admitted with AHF were included. On the day of hospital discharge, we measured the LUS and tracked these patients for up to 30 days to monitor emergency department visits, hospital readmission, and the number of days free from hospital stay.

Results

A total of 46 patients were included in the study. A composite outcome of ED visits or hospital readmission in the 30 days after hospital discharge was achieved for 22 (47.8%) patients. The LUS at hospital discharge had a receiver operating characteristic (ROC) area of 0.93 (95% CI, 0.82-0.99) to predict the composite outcome, against 0.67 (95% CI, 0.52-0.81) for the clinical congestion score (CCS). A LUS ≥ 7 at discharge had a sensitivity of 95.5% and a specificity of 87.5% to predict the composite outcome. The average exam duration was 176±65 (sd) seconds.

Conclusions

The LUS at hospital discharge following admission for AHF proves to be an accurate tool for predicting the likelihood of return to the ED and/or hospital readmission within 30 days post discharge.

## Introduction

Acute heart failure (AHF) significantly contributes to hospital admissions in medical wards. In the 30 days following the hospital discharge of these patients, the rate of return to the emergency department (ED) ranges from 23% to 52%, with more than half of these cases requiring hospital readmission. Typically, these readmissions occur within the first two weeks after hospital discharge, with the mean day being the 12^th^ day [1-4].

Lung ultrasound is already well-established as a highly sensitive method for detecting pulmonary interstitial edema and is superior to chest radiography [5-10]. The number of B-lines at hospital discharge after admission for AHF can predict mortality and hospital readmission in the upcoming months [11-18]. However, there is no standard method for counting the B-lines and thoracic area evaluated.

Quantitative methods for evaluating B-lines can be difficult to implement in daily practice. On the other hand, semi-quantitative methods are easier to perform and more likely to be implemented as part of a physical examination.

The lung ultrasound score (LUS) is a semi-quantitative method for the evaluation of lung deaeration. Learning this technique is straightforward, as evidenced by a previous study indicating that approximately 25 supervised exams are necessary for reproducibility [19]. In patients with acute respiratory distress syndrome, LUS can indicate disease severity and risk of death [20], detect lung aeration degree in patients in the prone position [21], and evaluate lung recruitment [22]. The LUS can also predict a good antibiotic response in patients with ventilator-associated pneumonia [23] and a risk of extubation failure [24].

Accordingly, we designed this study to investigate whether LUS on the day of discharge in patients admitted to the medical ward with AHF can predict the risk of returning to an ED and/or hospital readmission in the subsequent 30 days.

## Materials and methods

Patients selection

We conducted a prospective observational study at the medical ward of the emergency unit of the Clinics Hospital of the Ribeirao Preto Medical School, University of Sao Paulo, a tertiary university hospital in Ribeirao Preto, Sao Paulo, Brazil. The study was approved by the hospital ethics research committee, and all patients signed a free and informed consent form (approval number: 3.564.455). We conducted a pilot study with 20 patients admitted to the hospital with pulmonary or non-pulmonary diseases. Two experienced examiners with more than five years of experience in point-of-care ultrasound obtained the LUS separately to compare interobserver variability.

In the main study, we included all patients ≥ 18 years old who were admitted to the medical ward with a diagnosis of heart failure (as defined by the American Heart Association [25]), with AHF as the main or secondary cause of hospital admission, from September 2019 until July 2023. During the COVID-19 pandemic, the inclusion of patients was paused because the hospital became a reference center for severe cases. The exclusion criteria were pregnant women, patients with previous pneumectomy or lobectomy, burns in the thoracic region, and social conditions that did not allow follow-up. COVID-19 patients were also later added as an exclusion criterion.

Ultrasound findings and LUS

When the medical team decided on hospital discharge, one of the two examiners performed the ultrasound examination, evaluating the LUS and inferior vena cava (IVC).

**Figure 1:**
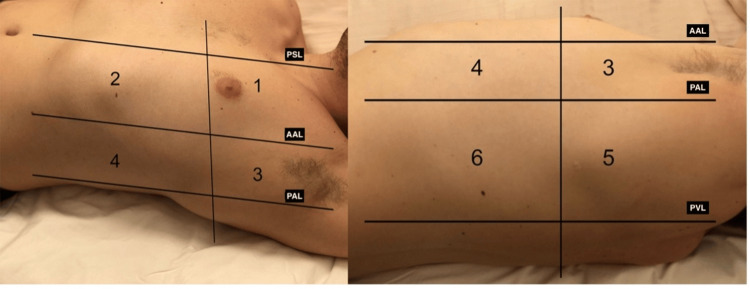
The six regions evaluated in each hemithorax for the LUS The vertical imaginary line follows the fifth intercostal space. The horizontal lines are the parasternal line (PSL), anterior axillary line (AAL), posterior axillary line (PAL), and paravertebral line (PVL). LUS: lung ultrasound score

For LUS, we chose the 12-region method (Figure 1). Each hemithorax was divided into six regions, delimited superiorly and inferiorly by the fifth intercostal space and laterally by the hemiclavicular line, and anterior and posterior axillary lines. A score from 0 to three was given to each region depending on the predominant finding: A score of 0 was given if lung sliding was present with A-lines or less than three B-lines inside each intercostal space; a score of one was given if there were three or more B-lines inside each intercostal space, originating from the pleural line or small subpleural consolidations; a score of two was given if coalescent B-lines were visible along all the intercostal space; and a score of three was given if there was a lung consolidation (tissue echogenicity and static or dynamic air bronchograms) and/or pleural effusion at the evaluated region. The exam was performed with the patient in a sitting position or, when not possible, in a semi-sitting position (bed elevation at 45º) and turning him/her sideways to perform the exam on the posterior regions.

The IVC was classified based on its maximum diameter and respiratory variation. The measurement was made approximately 3 cm from the entry to the right atrium or just caudal to the suprahepatic vein entry in the IVC, in the longitudinal plane, using B-mode. The IVC was considered “normal” if it had a maximum diameter of <2.1 cm and an inspiratory variation of >50%, “turgid” if it had a maximum diameter of ≥2.1 cm and an inspiratory variation of ≤50%, and “intermediate” if it met any criteria that did not fit previous classifications.

All the exams were performed using the phased array probe with one of the portable ultrasound machines (Esaote Mylab Five (Esaote SpA, Genoa, Italy) or GE Healthcare Logiq V2 (GE Healthcare, Waukesha, WI)) or the handheld probe (Butterfly iQ+, (Butterfly Network, Burlington, MA)).

Clinical data

The clinical congestion score on discharge day was obtained from the electronic health records and complemented by the examiner in person when pending information was necessary. We used the congestion score derived from the EVEREST trial [26], a score that ranges from 0 to 18, considering patients’ dyspnea, orthopnea, fatigue, jugular venous distension, rales, and edema.

Other data obtained from the patients were as follows: sex, age, type of heart failure (patients with heart failure with mild ejection fraction were included in the reduced ejection fraction group), comorbidities (coronary artery disease, diabetes, dyslipidemia, hypertension, smoking, alcoholism, obesity, chronic renal disease), total hospital stay length, medical ward stay length, serum creatinine on discharge day, cause of heart failure decompensation, patient origin before medical ward admission, and hospital admission in the 90 days before index admission.

We followed the patients up to 30 days after hospital discharge through telephone contact or data obtained from electronic health records.

First, we determined the LUS value with the best prognostic value to predict the composite outcome of return to an ED or hospital readmission in the 30 days following hospital discharge. The primary outcome was to evaluate the time free from hospital stay and time free from ED visit between the groups below and above the LUS cut-off.

The secondary outcomes were to evaluate the time to complete the LUS, to compare the group that met the composite outcome and the one that did not meet the composite outcome, the creatinine serum level on hospital discharge day, comorbidities, cause of heart failure decompensation, type of heart failure, IVC classification, and clinical congestion score, and to evaluate clinical congestion score accuracy to predict the composite outcome.

Data analysis

Categorical variables were expressed as percentages, quantitative variables as means, and standard deviations as measures of central tendency. In cases where a distribution with a non-normal pattern was observed or if there was an occurrence of discordant values (“outliers”) that could influence the measures of central tendency, the median and interquartile range were used. In situations where the variables present distributions with extreme deviations, the data were transformed for the variables that are included in the linear or logistic regression models.

To compare categorical variables, Fisher’s test was used for the difference between two groups and the chi-square test for the difference between several groups. For the comparison of unpaired continuous variables, a parametric Student's t-test was used when comparing the two groups. The comparison of paired continuous variables was performed using the same tests, applying the necessary correction. For the correlation between continuous variables, a parametric correlation test was used. When a non-parametric test was preferred because of the homogeneity of variance, the Kruskal-Wallis test was used.

To define the cut-off for the LUS value best related to the outcomes, receiver operating characteristic (ROC) curves were constructed. For the comparison between the time and the event, the Kaplan-Meier curves and the log-rank test were performed, and the Cox regression model was used for the multivariate analysis. For all tests used, it was considered statistically significant to have a value of p<0.05. Data analysis and construction of the graphs presented in the results were performed using the Stata 17® software (StataCorp LLC, College Station, TX).

## Results

The pilot study included 20 patients admitted to the medical ward or ED, with a median age of 59±16 years. The median LUS obtained was five. The Pearson's correlation coefficient was 0.9983, demonstrating very low interobserver variability.

The main study had 46 patients included. Twenty-two patients (47.8%) returned to an ED within 30 days after hospital discharge. Among these patients, the mean time to return to the ED was 18±8 days. Fourteen patients (30.4%) required hospital readmission. None of the patients died during follow-up. The average time to perform the LUS exam was 176±65 seconds, and there was no difference between those who met the composite outcome and those who did not, even with a higher score in the first group.

Comparing patients who met the composite outcome of return to the ED or hospital readmission within 30 days after hospital discharge, all the evaluated baseline characteristics were similar, except for the cause of cardiac decompensation and total length of hospital stay (Table 1). Infection was more common in patients who met the composite outcome (54.5% vs. 20.8%, p = 0.018), and acute coronary syndrome was more common in patients who did not meet the composite outcome (41.7% vs. 13.6%, p = 0.035).

**Table 1:** Baseline characteristics of the patients HFREF: heart failure with reduced ejection fraction; HFPEF: heart failure with preserved ejection fraction; STEMI: ST-elevation myocardial infarction; NSTEMI: non-ST-elevation myocardial infarction ☨ Heart failure with mid-range ejection fraction was included in this group.

Characteristics	Return to the ED or hospital readmission in 30 days (N = 22)	No return to the ED and/or hospital readmission in 30 days (N = 24)	Total (N=46)	p-value
Male sex (no. (%))	13 (59.1)	11 (45.8)	24 (52.2)	0.369
Age (years)	65±7	67±12	66±9.9	0.644
Heart Failure type (no. (%))				0.983
HFREF ☨	17 (77.3)	18 (75)	34 (76.1)	
HFPEF	5 (22.7)	6 (25)	11 (23.9)	
Comorbidities (no. (%))				
Coronary artery disease	13 (59.1)	13 (54.2)	26 (56.5)	0.736
Diabetes	15 (68.2)	11 (45.8)	26 (56.5)	0.127
Dyslipidemia	7 (31.8)	6 (25)	13 (28.3)	0.608
Systemic arterial hypertension	18 (81.8)	19 (79.2)	37 (80.4)	0.821
Smoking	6 (27.3)	3 (12.5)	9 (19.6)	0.207
Alcoholism	1 (4.76)	0 (0)	1 (2.2)	0.280
Atrial fibrillation	7 (31.8)	9 (37.5)	16 (34.8)	0.686
Obesity	8 (38.1)	5 (20.8)	13 (28.3)	0.202
Chronic kidney disease	10 (45.5)	9 (37.5)	19 (41.3)	0.584
Cause of decompensation (no. (%))				
Infection	12 (54.6)	5 (20.8)	17 (37.0)	0.018
Pneumonia	4 (18.2)	4 (16.7)	8 (17.4)	0.892
Urinary tract infection	2 (9.1)	1 (4.2)	3 (6.5)	0.499
Other infections	5 (22.7)	0 (0)	5 (10.9)	0.013
Poor treatment adherence	8 (36.4)	5 (20.8)	13 (28.3)	0.243
Acute coronary syndrome	3 (13.6)	10 (41.7)	13 (28.3)	0.035
Unstable angina	2 (9.1)	5 (20.8)	7 (15.2)	0.268
STEMI	0 (0)	3 (12.5)	3 (6.5)	0.086
NSTEMI	1 (4.6)	2 (8.3)	3 (6.5)	0.603
Other causes	4 (18.2)	4 (16.7)	8 (17.4)	0.892
Patient origin before admission to the medical ward (no. (%))				0.694
Emergency department	16 (72.7)	15 (62.5)	31 (67.4)	
Coronary care unity	3 (13.6)	5 (20.8)	8 (17.4)	
Semi-intensive care unity	0 (0)	1 (4.2)	6 (13.0)	
Intensive care unity	3 (13.6)	3 (12.5)	1 (2.2)	
Length of stay in the medical ward (days)	10±10	6±3	8±7	0.135
Total length of hospital stay: days	15±10	10±4	12±8	0.022
Recent hospital admission (no. (%))				0.115
≤30 days	0 (0)	4 (16.7)	4 (8.7)	
30-60 days	6 (27.3)	3 (12.5)	9 (19.6)	
61-90 days	0 (0)	1 (4.2)	1 (2.2)	

**Figure 2:**
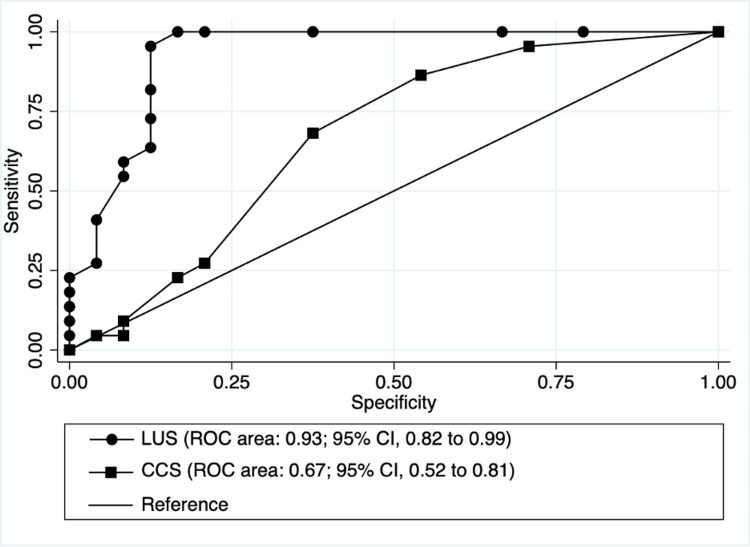
The ROC curves for the LUS and the CCS evaluating their sensitivity and specificity to predict the composite outcome of return to an ED or hospital readmission in the 30 days following hospital discharge from an AHF admission. CCS: clinical congestion score; CI: confidence interval; LUS: lung ultrasound score; ROC: receiver operating characteristic; AHF: acute heart failure

We evaluated LUS and clinical congestion score (CCS) at hospital discharge (Table 2). In the group of patients who met the composite outcome, the median LUS was 12±5 vs. 3±4 in the other group (p<0.001). The CCS did not meet statistical significance between groups (3±2 vs. 2±2, p=0.135). We conducted ROC curve analysis for LUS and CCS (Figure 2). The LUS had an ROC area of 0.93 (95% CI, 0.82 to 0.99), compared to an ROC area of 0.67 (95% CI, 0.52 to 0.81) for the CCS. A LUS ≥ 7 had a sensitivity of 95.5%, a specificity of 87.5%, a positive likelihood ratio of 7.64, and a negative likelihood ratio of 0.05 to predict the composite outcome.

**Table 2:** Outcomes LUS: lung ultrasound score; IVC: inferior vena cava ☨ The IVC was considered "normal" if it had a maximum diameter of <2.1 cm and an inspiratory variation of >50%, "turgid" if it had a maximum diameter of ≥2.1 cm and an inspiratory variation of ≤50%, and "intermediate" if it met any criteria that didn't fit previous classifications.

Outcome	ED return and/or hospital readmission in the 30 days after discharge (N = 22)	No ED return and/or hospital readmission in the 30 days after discharge (N = 24)	p-value
LUS at hospital discharge (score)	12±5	3±4	<0.001
LUS ≥ 7 at hospital discharge (no. (%))	21 (95.5)	3 (12.5)	<0.001
Exam duration (seconds)	167±41	185±82	0.947
IVC classification at hospital discharge (no. (%)☨)			0.035
Normal	3 (13.6)	8 (33.3)	
Intermediate	9 (40.9)	13 (54.2)	
Turgid	10 (45.5)	3 (12.5)	
Clinical congestion Score on discharge day (score)	3±2	2±2	0.135
Average furosemide dose used at the medical ward (mg/d)	73.8±50.7	54.9±34.2	0.143
Creatinine at hospital discharge (mg/dl)	1.80±0.7	1.47±0.8	0.132
Ultrasound device used (no. (%))			0.595
Esaote Mylab Five	1 (4.6)	3 (12.5)	
GE Healthcare LogiqV2	15 (68.2)	16 (66.7)	
Butterfly iQ+	6 (27.3)	5 (20.8)	

**Figure 3:**
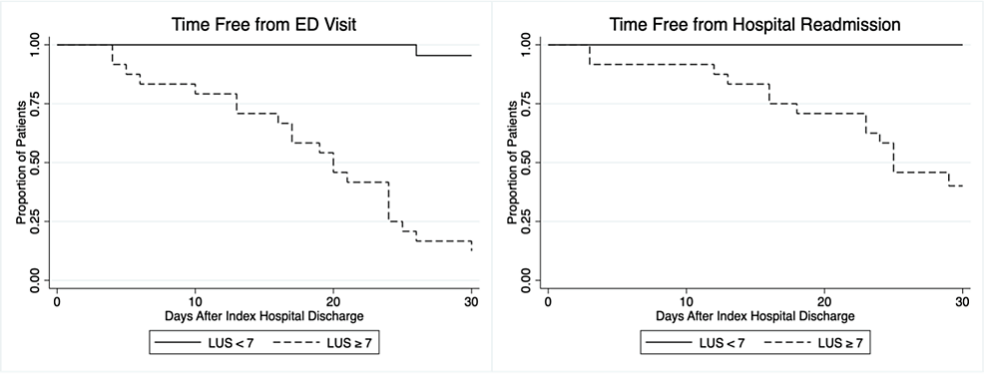
Kaplan-Meier curves comparing time free from ED visit in the 30 days following hospital discharge (on the left) and time free from hospital stay for patients who were readmitted (on the right) with LUS at hospital <7 (continuous line) or ≥7 (dashed line). ED: emergency department; LUS: lung ultrasound score

Considering the LUS cut-off ≥ 7 derived from the ROC curve, 95.5% (95% CI 77.1-99.8%) of the patients met the composite outcome against 12.5% (95% CI 2.6-32.3%) of the patients in the other group (p<0.001). The Kaplan-Meier curve showed a significant difference in the time free of hospital stay between groups (log-rank test p<0.001) (Figure 3).

The IVC classification showed a statistically significant difference between those patients who met the composite criteria and those who did not. In the first group, it was classified as normal, intermediate, or turgid in 13.6%, 40.9%, and 45.5%, respectively, against 33.3%, 54.2%, and 12.5% in the latter one (p = 0.035). The creatinine levels at hospital discharge, the ultrasound device used, and the length of stay in the medical ward were not different between the groups. However, the total length of hospital stay, including days before admission to the medical ward, was significantly higher in the group that met the composite outcome (15±10 days versus 10±4 days, p=0.022) (Table 2).

We performed a post hoc analysis comparing all the variables that were statistically different between the two groups to observe if LUS at hospital discharge was still related to the composite outcome. We ran a logistic regression model that considered CCS, IVC classification, total length of hospital stay, infection, and acute coronary syndrome as causes of decompensation. The LUS at hospital discharge was still independently related to the composite outcome (OR 1.5, 95% CI 1.16-1.95).

Analyzing the data on how LUS behaves in each region of the thorax, we tabulated the proportion of patients who received a score from 0 to three in each of the 12 thoracic regions evaluated in the study, grouped among those who presented the composite outcome of return to an ED or hospital readmission within 30 days after hospital discharge and those who did not (Table 3). When evaluated separately, the thoracic regions that showed a correlation with the composite outcome were the right lateral inferior (p<0.001), right posterior superior (p=0.003), right posterior inferior (p<0.001), left lateral superior (p=0.01), left lateral inferior (p<0.001), left posterior superior (p=0.001), and left posterior inferior (p<0.001).

**Table 3:** Proportion of patients who received a score of 0, one, two, or three on LUS for each one of the 12 thoracic regions evaluated ED: emergency department; LUS: lung ultrasound score; LAS: left anterior superior; LAI: left anterior inferior; LLS: left lateral superior; LLI: left lateral inferior; LPS: left posterior superior; LPI: left posterior inferior; RAS: right anterior superior; RAI: right anterior inferior; RLS: right lateral superior; RLI: right lateral inferior; RPS: right posterior superior; RPI: right posterior inferior

Patients who did not return to the ED or were readmitted 30 days after discharge											
Left hemithorax	Region	LUS 0	LUS 1	LUS 2	LUS 3	Right hemithorax	Region	LUS 0	LUS 1	LUS 2	LUS 3
	LAS	100%	0%	0%	0%		RAS	100%	0%	0%	0%
	LAI	95.8%	4.2%	0%	0%		RAI	91.7%	8.3%	0%	0%
	LLS	91.7%	4.2%	4.1%	0%		RLS	87.5%	12.5%	0%	0%
	LLI	58.3%	29.2%	4.2%	8.3%		RLI	54.2%	41.7%	0%	4.1%
	LPS	91.7%	8.3%	0%	0%		RPS	87.5%	12.5%	0%	0%
	LPI	58.3%	29.2%	4.2%	8.3%		RPI	58.3%	29.2%	4.2%	8.3%
Patients returned to the ED or were readmitted 30 days after discharge											
Left hemithorax	Region	LUS 0	LUS 1	LUS 2	LUS 3	Right hemithorax	Region	LUS 0	LUS 1	LUS 2	LUS 3
	LAS	90.9%	9.1%	0%	0%		RAS	90.9%	9.1%	0%	0%
	LAI	90.9%	9.1%	0%	0%		RAI	63.6%	27.3%	9.1%	0%
	LLS	54.6%	40.9%	4.5%	0%		RLS	59.1%	31.8%	9.1%	0%
	LLI	0%	27.3%	50.0%	22.7%		RLI	4.6%	13.6%	40.9%	40.9%
	LPS	36.4%	54.6%	4.5%	4.5%		RPS	36.4%	40.9%	9.1%	13.6%
	LPI	0%	18.2%	45.4%	36.4%		RPI	0%	27.3%	31.8%	40.9%

We also tested if a simplified version of the LUS excluding posterior regions on both sides (an eight-region score that goes from zero to 24) would maintain its accuracy compared with the 12-region one. The ROC area was only 0.518 (95% CI, 0.369-0.671), with a very limited prognostic value.

## Discussion

The LUS is a feasible tool to be added to the physical examination of our patients with AHF when an ultrasound machine is available. Our study showed that it has low interobserver variability and adds an acceptable amount of time to the traditional exam, which is lower than previously demonstrated [19]. When performed on the day of hospital discharge in patients admitted with AHF, it can accurately predict the risk of return to the ED and/or hospital readmission in the following 30 days, performing better than the CCS. 

Based on our study, 50% of the patients who returned to an ED 30 days after hospital discharge did so around day 20. Implementing a follow-up return two weeks after hospital discharge of these patients with LUS ≥ 7 could detect those with early signs of heart failure decompensation and prevent hospital readmissions and unscheduled ED visits. The LUS-HF study [27] demonstrated that lung ultrasound-guided follow-up improves clinical outcomes.

Our study has some limitations. First, it was a unicenter study conducted in a tertiary university hospital. Therefore, the profile of patients admitted to the medical ward can be more severe than in other hospitals. Although following international guidelines for the treatment of AHF, the medications used and their dosage varies greatly based on patients’ individual characteristics, and we have not obtained data on medications used by patients at hospital discharge. In addition, our medical ward is situated in a tertiary emergency center, which may influence a greater pressure for early discharge as soon as clinical and laboratory improvement is achieved due to the high inflow of patients. This may reflect the high number of returns to an ED and hospital readmissions we found, although this is similar to what Shammas et al. (2018) found in their study [2].

Second, we had a small sample size. We opted not to include COVID-19 patients, and as we became a reference center for severe cases, that slowed our inclusion of patients. Interestingly, LUS can predict outcomes in patients admitted to the ED with COVID-19 [28]. On the other hand, we opted not to exclude from our study patients with other types of pulmonary diseases associated with the AHF that could also influence LUS (like pneumonia, pulmonary fibrosis, etc.) since these diseases can also influence outcomes and to keep the study design closer to real practice.

Third, we opted not to include other measurements of intracardiac pressure elevation. The brain natriuretic peptide (BNP) at hospital discharge has already demonstrated good predictive power for poor outcomes such as death or rehospitalization in the one-year follow-up [29] and has a good correlation with the total number of B-lines on lung ultrasound at hospital discharge [12, 13]. Besides that, it adds cost to the hospital stay and is not available everywhere, and as the LUS-HF study [28] showed, we can improve the outcome. The same applies to other measurements on the echocardiogram, such as pulmonary artery pressure, E/e’. We wanted to keep our study design as close to real practice as possible.

Fourth, AHF can be a newly arisen diagnosis (“de novo”) or an acutely decompensated chronic heart failure, with the latter having the worst prognosis [30]. We have not specified in our study the type of AHF our patients had. This can partially explain why our patients with acute coronary syndrome as the cause of admission had fewer readmissions and returns to the ED compared with other causes of cardiac decompensation.

We need more studies to affirm that a hospital treatment guided by LUS could improve these patients’ LUS at discharge and improve outcomes. We also need more studies to evaluate what is the best treatment to improve LUS since we have different profiles of AHF and clinical congestion and hemodynamic congestion do not always improve simultaneously.

## Conclusions

The 12-region LUS on the day of hospital discharge can independently predict the likelihood of returning to the ED or hospital readmission within 30 days following a hospital admission for AHF. Notably, it demonstrates superior performance compared with the clinical congestion score. A LUS ≥ 7 at hospital discharge had better sensitivity and specificity to detect a 30-day return to the ED and hospital readmission. The LUS exhibits low interobserver variability and requires an additional approximately three minutes to the traditional examination. Further studies are warranted to assess whether LUS serves solely as a prognostic marker or if it could be a modifiable factor in AHF admissions capable of influencing outcomes.
